# Refraction-Assisted Solar Thermoelectric Generator based on Phase-Change Lens

**DOI:** 10.1038/srep27913

**Published:** 2016-06-10

**Authors:** Myoung-Soo Kim, Min-Ki Kim, Sung-Eun Jo, Chulmin Joo, Yong-Jun Kim

**Affiliations:** 1 Department of Mechanical Engineering, Yonsei University, 50 Yonsei-ro, Seodaemun-Gu, Seoul, Republic of Korea

## Abstract

Solar thermoelectric generators (STEGs), which are used for various applications, (particularly small size electronic devices), have optical concentration systems for high energy conversion efficiency. In this study, a refraction-assisted STEG (R-STEG) is designed based on phase-change materials. As the phase-change material (PCM) changes phase from solid to liquid, its refractive index and transmittance also change, resulting in changes in the refraction of the sunlight transmitted through it, and concentration of solar energy in the phase-change lens. This innovative design facilitates double focusing the solar energy through the optical lens and a phase-change lens. This mechanism resulted in the peak energy conversion efficiencies of the R-STEG being 60% and 86% higher than those of the typical STEG at solar intensities of 1 kW m^−2^ and 1.5 kW m^−2^, respectively. In addition, the energy stored in PCM can help to generate steady electrical energy when the solar energy was removed. This work presents significant progress regarding the optical characteristic of PCM and optical concentration systems of STEGs.

Solar energy is a clean and sustainable source of renewable energy; consequently, harvesting solar energy is one of the most attractive methods for overcoming energy shortage. Currently, there are two means for directly converting solar energy into electricity: photovoltaic technology, which converts photon energy into electricity via electron–hole pair generation, and thermoelectric technology, which converts temperature differences into electric potential based on the Seebeck effect. Compared with solar cells, solar thermoelectric generators (STEGs) currently have low energy conversion efficiency but also possess a variety of advantages—i.e., good stability, high reliability, long operation life, environmental friendliness, and silent operation^1^,^2^,^3^,^4^. Additionally, STEGs possess the advantage of being able to utilize a larger portion of the solar spectrum^5^.

The energy conversion efficiency of thermoelectric generators (TEGs) has significantly improved in recent times. The major determinant of the energy conversion efficiency of TEGs are the operating temperature and the materials’ dimensionless figure of merit (ZT), defined as ZT = α2T/κρ, where α is the Seebeck coefficient, κ is the thermal conductivity, and ρ is the electrical resistivity^6^,^7^,^8^. The theoretical maximum efficiency of TEGs (ηTEGs) can be written as a function of ZT and the temperature difference between the hot junction (Th) and cold junction (Tc)^8^:


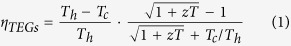


According to Equation (1), an energy conversion efficiency of approximately 1% can be reached by applying a temperature difference of 20 °C across telluride alloys used in STEGs with ZT = 1 and Tc = 25 °C^9^. In recent years, superlattice- and quantum-dot–based thermocouples with room temperature ZT values as high as 2.5 have been demonstrated^10^,^11^. However, commercial materials with ZT = 1 are not yet available; these material limitations are the primary concern for practical applications^12^. Another method that can improve the energy conversion efficiency is the creation of a significant temperature difference across the TEGs with the solar radiation flux.

Various methods for creating a larger temperature difference in STEGs have been proposed. One common STEG design utilizes an optical system to collect sunlight on the hot junction of the TEG. In 1954, Telkes used a 50× optical concentration with a lens to achieve 3.3% efficiency^13^. Li *et al*. designed a prototype optical-concentrated STEG and evaluated its performance using a numerical method. Their results showed that 14.1% is the highest possible efficiency of a TEG that employs LAST (AgPbmSbTe2+m) alloys as the thermoelectric material^3^. Fan *et al*. reported STEGs constructed using a parabolic dish concentrator. Under maximum heat flux, a TEG can generate 4.9 W, corresponding to 2.9% efficiency^14^. Another approach for creating the temperature difference across the STEG is the use of a selective absorber configuration that exploits thermal concentration. Kraemer *et al*. designed an STEG with a flat-panel selective absorber; the designed STEG showed a peak efficiency of 4.6% under AM1.5G conditions^15^. Cao *et al*. developed a thin film selective absorber based on double cermet layer and antireflection coating. Their results showed that the selective absorber coating has the ability to absorb over 90% of the sunlight^16^. A different approach uses heat storage to generate the temperature difference that drives an STEG, even in the absence of an energy source. Agbossou *et al*. and Zhang *et al*. designed a prototype STEG attached to heat storage filled with a phase-change material (PCM) and evaluated its performance using a numerical method^1^,^17^. They showed that the STEG was capable of producing power at night using the stored heat in the PCM.

As stated above, a key challenge in solar thermoelectric power conversion is the method of creating a significant temperature difference across the TEG with only a low solar radiation flux. Thus, concentrating as much sunlight as possible into one point is very important. Furthermore, the actual solar radiation flux varies according to time and location. Therefore, in order to improve the efficiency of STEGs, the impact of environmental conditions needs to be mitigated. When solar energy is strong, it is necessary to increase the production of energy in order to collect the maximum solar radiation flux. There is also a need for sustained energy generation even when solar energy is weak. Reusing dissipated heat through a PCM is one possible solution to the above problem. Among the various thermal energy-storage methods, PCMs are the best effective owing to their thermal energy storage density and isothermal operating characteristics^18^,^19^,^20^,^21^,^22^. PCMs can store large amounts of heat energy during phase-change. Subsequently, the TEGs combined with PCM can generate electricity from the stored heat energy without energy source^23^. In addition, as the PCM changes phase from solid to liquid, the refractive index and transmittance of the material also change. These phenomena change the refraction of transmitted sunlight through the PCM. This may be another way to concentrate sunlight without special active devices.

In this study, we investigated solar energy harvesting by coupling a TEG and a phase-change lens that was produced by the solar power melting the PCM. We verified that the PCM could act as a lens by measuring the light transmittance and the refractive index of the PCM in accordance with the phase change. Then, we developed a refraction-assisted STEG (R-STEG) consisting of a Polydimethylsiloxane (PDMS) dome-shaped lens, PCM, wavelength-selective absorber, and a TEG module. The developed R-STEG first focuses the solar energy through the PDMS dome-shaped lens. Then, it re-concentrates the energy via the liquid PCM lens based on the phase-change lensing effect. As a result, the temperature differences between the hot and cold junctions increase more than a typical STEG with a single lens. The energy conversion efficiencies of the developed R-STEG were found to be 60% and 86% higher than those of a typical STEG at solar intensities of 1 kWm^−2^ and 1.5 kWm^−2^, respectively. In addition, the R-STEG generated energy for a period of time even when the energy source was removed. Furthermore, we verified that the R-STEG was able to successfully convert solar energy into electrical energy and generate energy for part of the night in an ambient open environment.

## Results

### Optical Analysis of PCM

**Figure 1:**
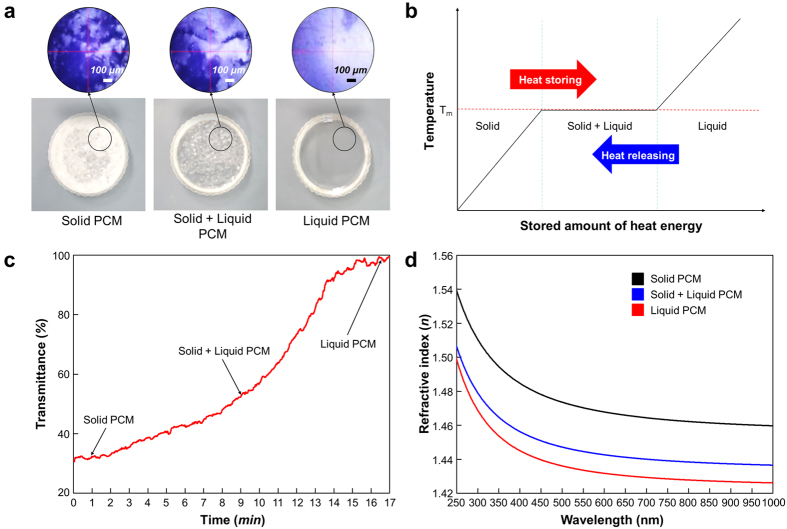
Optical characteristics of the phase-change material (n-octadecane). (**a**) Solid, solid–liquid, and liquid PCM and the crystalline structure of each phase. (**b**) Temperature changes during phase-change energy storage. (**c**) Transmittance changes with respect to phase of the PCM. (**d**) Spectral refractive indices of the PCM with respect to the crystalline structure of the PCM.

As stated earlier, a PCM has a high latent heat of fusion, which enables it to store and release large amounts of energy. When the phase of a material changes from solid to liquid and vice versa, latent heat is absorbed or released at an almost constant temperature, as shown in Fig. 1(b). The latent heat of fusion during the phase change plays an important role in energy storage. Compared to sensible heat storage, latent heat has several times more energy storage and release capacity. From among various PCMs, n-octadecane (Octadecane, Sigma-Aldrich)—a type of paraffin wax—was used in this study. N-octadecane has a large latent heat of fusion of approximately 244 J·g^−1^ and a low melting point less than 29 °C. Consequently, phase changes can easily occur even with small solar energy, and high energy savings are possible compared to other materials. In addition, it has the advantages of being low cost and having very low volume changes during melting.

The PCM discussed has been used and studied mainly as energy-storage material. However, as a PCM changes phase from solid to liquid, its refractive index and transmission efficiency also change. These phenomena change the refraction of transmitted sunlight through the PCM, which may consequently prove to be another way to concentrate sunlight without special active devices. Two factors primarily affect lens performance: (1) light transmittance, which is light transmission through the medium without being absorbed or scattered, and (2) refractive index, which describes how light, or any other radiation, propagates through that medium. When light passes through a medium, energy loss through absorption or dispersion occurs. If the light transmittance of the medium is low, the energy loss will be high, which has a significant influence on the efficiency of the lens. In addition, the more the light refracts, the better the light will be concentrated. Therefore, to satisfy the above essential conditions, the medium must have both high light transmittance and a high refractive index. In general, Poly(methyl methacrylate) (PMMA), which has a transmittance above 92% and refractive index above 1.48, is commonly used as a lens material.

To evaluate the optical characteristics of the PCM (n-octadecane), we measured its light transmittance and refractive index. We measured its optical characteristics in real time according to the phase change of the PCM during application of a 1.5 kW m^−2^ light source. Figure 1(a) shows the solid, solid–liquid, and liquid state PCM along with the corresponding crystalline structure in each state. While changing to the solid state from the liquid state, the PCM crystallizes with a change in transparency (See Supplementary movie S1). The solid-state PCM has a rough, translucent surface because of the crystalline structure. The light transmittance of the PCM is approximately 35% at this time. When heating changes the phase of the PCM from a solid to a liquid, the polycrystalline structure is broken, so that the light transmittance is increased. At the end of the phase change, the liquid state of the PCM has a light transmittance of 90% or more, as shown in Fig. 1(c). Furthermore, because the crystalline structure changes in accordance with the state of the PCM, the refractive index also varies in accordance with the state of the PCM. To evaluate the refractive index for each state of the PCM, we measured the refractive index using an ellipsometer (RC2, J.A. Woollam Co.) in the wavelength range 250 nm to 1000 nm. The results showed that the refractive index of the PCM changed with changes in the crystalline structure of the PCM, as shown in Fig. 1(d). The PCM had the highest refractive index in the solid state. In the liquid state, it had a refractive index ranging from 1.5 at 250 nm to 1.43 at 1000 nm. The results of the evaluation of the optical properties of the PCM indicated that the liquid PCM has the highest light transmittance and a good refractive index. These optical properties are similar to those of conventional lens materials such as PMMA and allyl diglycol carbonate (CR-39). Through the above results, we confirmed that the liquid PCM could act as a lens in solar power generation. We hereafter call these phenomena the ‘phase-change lensing effect’.

### Refraction-Assisted Solar Thermoelectric Generator Setup

**Figure 2:**
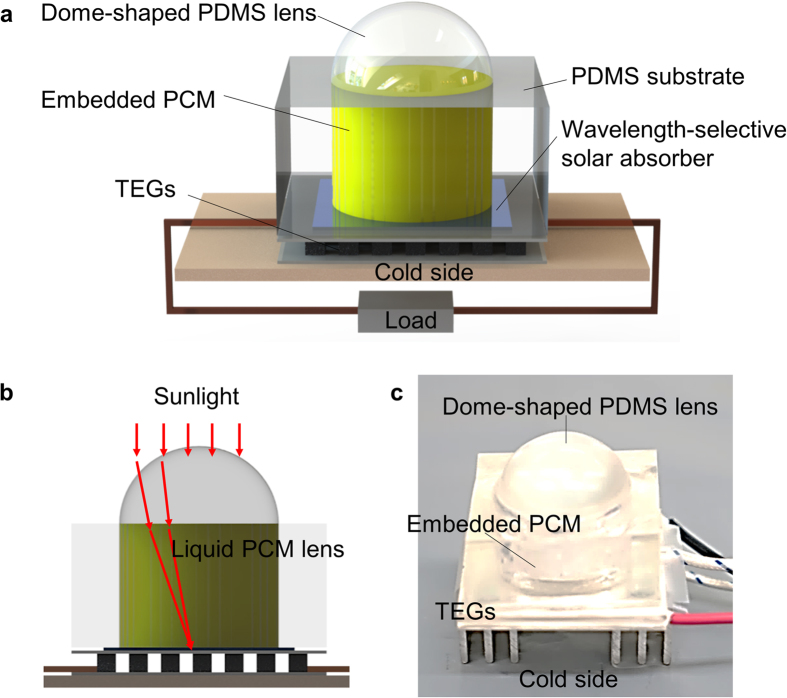
Structure of the refraction-assisted STEG. (**a**) A refraction-assisted STEG comprises three subsystems: (i) An optical system (dome-shaped PDMS lens); (ii) a phase-change system (PDMS container, PCM (n-octadecane)); and (iii) an energy generation system (wavelength-selective solar absorber, TEG module, and cooling part). (**b**) Illustration of the sunlight concentration through the optical lens and the liquid PCM lens. (**c**) A photograph of the device.

A refraction-assisted STEG is composed of several subsystems: an optical system that concentrates the solar radiation and an energy generation system comprising a wavelength-selective solar absorber, TEG module, and cooling part. Furthermore, a phase-change system is placed between the optical concentration system and the energy generation system, as shown in Fig. 2. The phase-change system is configured with PCM and PDMS molds to carry the PCM. As shown in Fig. 2(b,c), the phase-change system acts as a second lens to refocus the solar radiation in addition to heat storage provided by the phase-change of the PCM. The refraction-assisted STEG improves its energy conversion efficiency by concentrating the solar energy and producing energy with the stored energy for some time after sunset. This simple mechanism is successfully employed to improve the solar energy conversion efficiency without additional energy consumption.

### Working Mechanism

**Figure 3:**
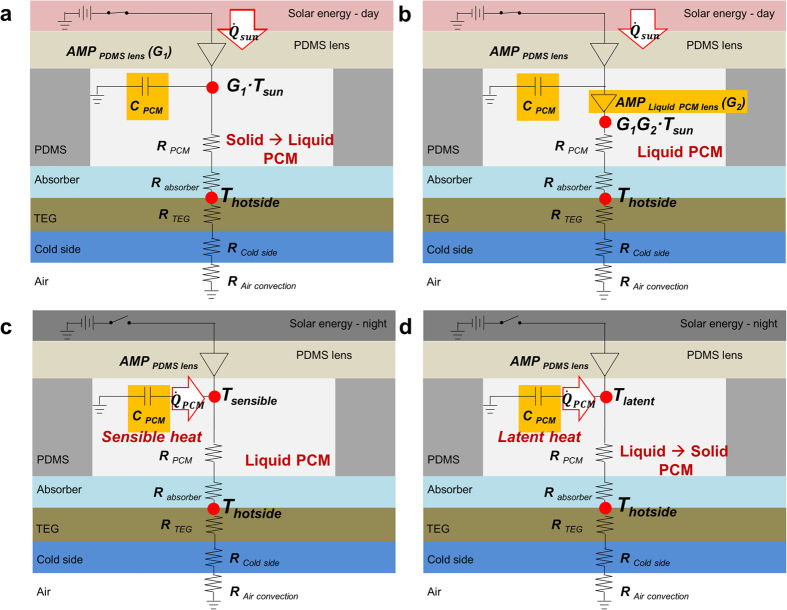
Working mechanism of the refraction-assisted STEG. (**a**) Solar energy generation with energy storage based on the phase-change of the PCM in daytime. (**b**) Assistance of the phase-changing lens to refocus the solar energy in the daytime. (**c**) Energy generation via release of the sensible heat stored in the liquid PCM. (**d**) Energy generation via release of the latent heat stored in the PCM

The electricity generation process in a refraction-assisted STEG is based on the phase-change lensing effect and the energy-storage ability of the PCM. The working mechanism can be explained more clearly using the simplified equivalent thermal circuits illustrated in Fig. 3. Four equivalent thermal circuits are presented in order of precedence. Figure 3(a) shows the first step of solar energy harvesting with the phase change of the PCM in the daytime. When solar energy is applied to the TEG, it is first concentrated by the dome-shaped PDMS lens. Then, the PCM changes state from solid to liquid by absorbing the concentrated energy as heat. During the phase-change operation, the internal temperature of the PCM is constant. The PCM functions as a capacitor and a resistor in the thermal circuit. As a result, the temperature of the hot side of the TEG module increases according to the sum of the amount of primarily concentrated energy and the temperature of the PCM (see Supplementary Information).

**Figure 4:**
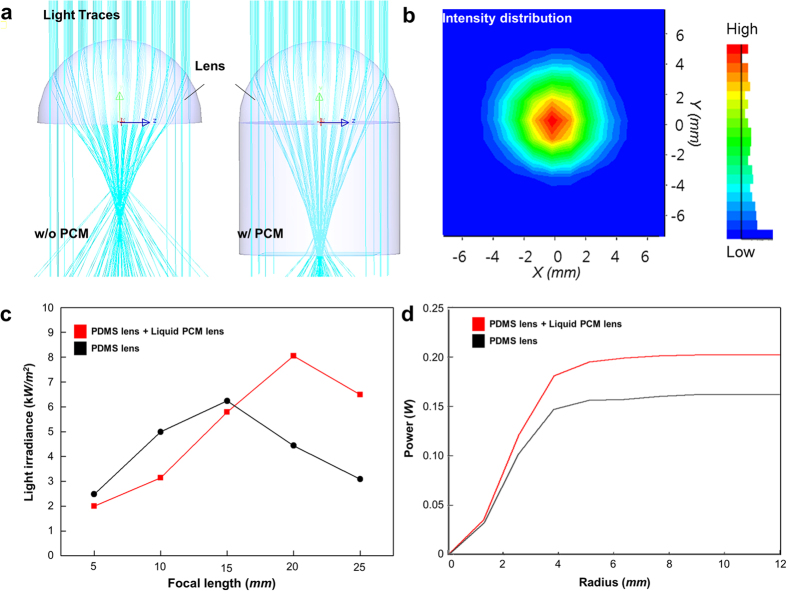
Results of the computational simulation. (**a**) Light traces depending on the presence or absence of the liquid PCM lens. (**b**) Light intensity distribution at the absorber, after light passes through the lens. (**c**) Light irradiances of each optical lens according to the focal length. (**d**) Total power reaching the absorber according to the radius of the absorber.

The second step is the assistance of the phase-changing lens to refocus the solar energy in the daytime, as shown in Fig. 3(b). At the end of the phase change, the liquid PCM functions as a second lens because of the phase-change lensing effect. The energy concentrated by the PDMS lens is refocused through the liquid PCM lens, which operates as an amplifier in the thermal circuit. This mechanism helps to improve the energy conversion efficiency of the solar generator. To confirm the phase-change lensing effect, computational simulations were conducted using Lighttools 8.3. Figure 4(a) depicts the results of light traces depending on the presence or absence of the liquid PCM lens. These results show that the liquid PCM lens helps concentrate more light by guiding the way of light. The maximum light irradiance of the lens model with liquid PCM lens was 29% higher than that without, as shown in Fig. 4(c,d). The efficiency of the refraction-assisted STEG (*η*_R-STEG_) in the daytime can be approximately expressed as the product of the optical lens thermal absorber efficiency (*η*_opth_) and the thermoelectric device’s efficiency (*η*_TEGs_): (see Supplementary information)


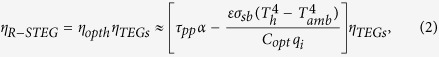


where *τ*_pp_ is the total transmittance of the PDMS and the liquid PCM, α is the absorptance, *ε* is the effective emittance of the wavelength-selective solar absorber, *σ*_*sb*_ is the Stefan-Boltzmann constant, *C*_*opt*_ is the optical concentration of the dome-shaped PDMS lens and the liquid PCM lens, *T*_*h*_ is the hotside temperature of the TEG, *T*_*amb*_ is the ambient temperature, and *q*_*i*_ the incident solar flux. The above expression shows that the optical lens thermal absorber efficiency increases with increasing *C*_*opt*_ and decreases with increasing *T*_*h*_; however, the thermoelectric device’s efficiency (Equation (1)) increases with increasing *T*_*h*_. Taking typical values (*τ*_pp_ = 0.94, α = 0.95, *ε* = 0.05, *q*_*i*_ = 1 kW m^−2^, *zT* = 1, *T*_*amb*_ = 25 °C) and the above simulation results, the respective efficiencies of the refraction-assisted STEG and a typical STEG without the PCM lens system are approximately 7.4% and 6.1%, respectively. The theoretical efficiency of the refraction-assisted STEG is approximately 22% higher than that of the typical STEG.

The final step is the production of energy using the energy stored in the PCM. At night, it is impossible to produce energy directly based on solar energy. Therefore, the stored energy is released via the phase change of the PCM from liquid to solid, as shown in Fig. 3(c,d). The temperature of liquid PCM drops to its freezing point with the release of sensible heat. When PCM changes phase from liquid to solid, the PCM temperature remains constant by releasing of the latent heat. The direction of the heat flux emitted from the PCM is the same as that in the daytime, because the PCM is located on the TEG. Therefore, it is possible to produce energy without additional conversion circuitry. At the end of the phase change, the PCM stops emitting heat and energy generation ceases.

### Performance Comparison between the R-STEG and a Typical STEG

The output performances of the refraction-assisted STEG and that of a typical STEG were examined under illumination intensities corresponding to 1 kW m^−2^ and 1.5 kW m^−2^. The experiment involved measuring the hot side (wave-selective solar absorber) temperature, cold side (heat sink) temperature, and the closed-circuit voltage over time. Then, the temperature differences between the hot and cold sides, the output power, and the energy production were calculated based on the measured results. The ambient temperature during the experiment was 25 °C. At the start of the experiment, light was applied for 30 min from the end of the phase change.

**Figure 5:**
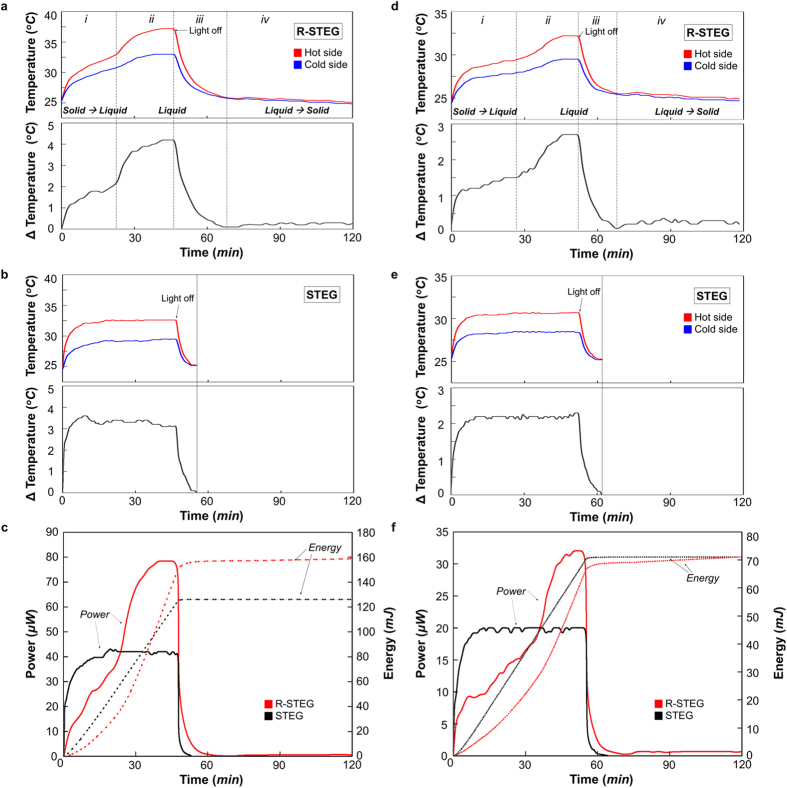
Performance comparison of refraction-assisted STEG (R-STEG) and typical STEG (STEG) at incident solar radiation fluxes of (**a**–**c**) 1.5 kW m^−2^ and (**d**–**f**) 1 kW m^−2^. (**a**,**b**,**d**,**e**) Temperature change and temperature difference of the R-STEG and STEG. (**c**,**f**) Comparison of the output power and energy of the R-STEG and STEG.

Figure 5(a,b) show the temperature change of the refraction-assisted STEG (R-STEG) and the typical STEG (STEG) under illumination intensities corresponding to 1.5 kW m^−2^. The temperature profile of the R-STEG can be divided into four sections—Fig. 5(a–i), (ii), (iii), and (iv)—in accordance with the above working mechanism. The first and second sections show the temperature profile when there is a light source, the third and fourth sections show the temperature profile when there is no light source. When light was applied to the R-STEG, the temperature rapidly increased up to the melting point, then remained virtually constant (Fig. 5(a-i)). In the second section (Fig. 5(a-ii)), the liquid PCM lens further concentrates the energy, and the temperature of the PCM rises simultaneously. The temperature of the hot side again rises rapidly because of these phenomena. The maximum temperature of the hot side was 37.2 °C, and the temperature difference between the hot and cold sides was 4.2 °C. Following removal of the light source, the sensible heat stored in the PCM was emitted to the freezing point. Therefore, the temperature change progressed slowly, as shown in the third section (Fig. 5(a-iii)). The final section (Fig. 5(a-iv)) shows the temperature change resulting from the release of the latent heat of the PCM after it starts to solidify. The temperature difference between the hot and the cold junctions steadily remained between 0.5 °C and 0.4 °C. The temperature profile of the STEG is relatively simpler than that of the R-STEG. When the light was applied to the STEG, the temperature increased to the thermal equilibrium state. The maximum temperature of the hot side was 32.4 °C and the temperature difference between the hot and cold sides was 3.1 °C. Following removal of the light source, the temperature dropped sharply within 7 min. The results show that the temperature difference between the hot and cold sides of the STEG is higher than that of the R-STEG until the liquid PCM lens is generated. Following generation of the liquid PCM lens, the temperature difference of the R-STEG becomes 35% higher than that of the STEG. These temperature profiles are clearly reflected in the closed-circuit voltage and current (see Supplementary Fig. S1(a,b)).

Figure 5(c) shows the output power and energy of the R-STEG and STEG under the change in temperature in Fig. 5(a,b). These results show that the output power of R-STEG is lower than that of STEG until the liquid PCM lens is generated. At the end of phase change, the output power of R-STEG increased rapidly. The peak powers of the R-STEG and STEG are 78.4 μW and 42 μW, respectively. The average output power of the R-STEG and STEG are 44.1 μW and 35.7 μW, when light was applied to each device. The average output power of the R-STEG based on energy stored in the PCM was approximately 0.6 μW, and the amount of energy produced was approximately 10 mJ. The total energy produced by each device during the experimental two hours was 160 mJ for the R-STEG and 126 mJ for the STEG. In these experiments, the experimental time was limited to 30 min after the end of the phase change. However, time which is able to harvest solar energy has a range of about 3 to 6 hours depending on the altitude and microclimate of a location^24^. Therefore, the average power of the R-STEG becomes larger than that of the STEG with increasing energy harvesting time, and accordingly the amount of produced energy also increased.

Additional experiments were conducted in a similar manner using illumination intensities corresponding to 1 kW m^−2^, as shown in Fig. 5(d–f). For this illumination intensity, the respective maximum powers of the R-STEG and STEG were 32 μW and 20 μW. The average output powers of each device were about 16 μW respectively, while the light source was turned on. The average output of the R-STEG based on energy stored in the PCM was approximately 0.6 μW, and the amount of produced energy was approximately 4.5 mJ. The total amount of produced energy by each device during the experimental time of two hours was 72 mJ for the R-STEG and 71 mJ for the STEG.

### Demonstration in an Open Environment

**Figure 6:**
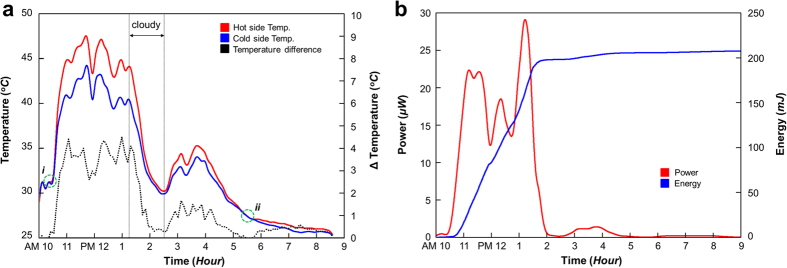
Demonstration of the refraction-assisted STEG (R-STEG) in an open environment. (**a**) Temperature of the R-STEG along the experimental timeline. (**b**) Output power and energy of the R-STEG along the experimental timeline.

To demonstrate the performance of the R-STEG in an ambient environment, we performed an experiment outdoors, as shown in Fig. 6. The experiment was performed from 10 AM until the end of energy production. The ambient temperature changed from 28 °C to 31 °C with various natural wind speeds during the experiment. Because STEGs produce low power, cooling and tracking systems, which consume additional power, were not attached in this experiment. Fig. 6(a) shows the temperature profiles of the STEG. The PCM completed its phase change from solid to liquid after 30 min into the experiment (Fig. 6(a–i)), and the temperature then increased rapidly. The temperature changed in response to variations in the solar radiation flux. The maximum temperature difference between the hot and cold sides was 5.4 °C. The solidification of the PCM occurred at the freezing point (Fig. 6(a-ii)), and the latent heat generated a temperature difference of approximately 0.5 °C for 3 h. Supplementary Fig. S2 shows the electrical performance of the R-STEG with a maximum closed-circuit voltage of 6.5 mV and a current of 5.4 mA. The energies generated using solar and stored latent heat as the energy source were 200 mJ and 10 mJ, respectively as shown in Fig. 6(b). The result shows that the R-STEG can generate approximately 5% additional energy after cessation of solar radiation flux. These demonstrations confirm that the R-STEG can operate in an open environment under a variety of conditions.

## Discussion

We have shown that the liquid PCM lensing effect can be effectively employed to simultaneously induce concentration of solar radiation flux and provide heat storage. To evaluate the optical characteristics of the PCM (n-octadecane) for the optical lensing effect, we measured the light transmittance and refractive index of the PCM. The liquid PCM has a refractive index ranging from 1.5 to 1.43 and light transmittance index of at least 95% . The refraction-assisted STEG can improve the energy conversion efficiency by utilizing these optical characteristics: the liquid PCM improves the energy concentration rate by changing the refraction of solar light. Additionally, the PCM stores and releases energy during the phase-change.

The maximum output powers of the R-STEG were 60% and 86% higher than those of the typical STEG at solar intensities of 1 kW m^−2^ and 1.5 kW m^−2^, respectively. The average output power of the STEG produced by the latent heat stored in the PCM was approximately 0.6 μW. The open environment test shows that the R-STEG can generate approximately 5% additional energy after cessation of solar radiation flux. However, despite significant increase of maximum output power and energy production, the R-STEG has still insufficient performance to supply power to small size electronic devices. The R-STEG can improve the energy conversion efficiency through various researches. This analysis of the R-STEG does not consider heat transfer efficiency and heat loss in the structure and materials. To further enhance its performance, the heat transfer and heat loss problems need to be addressed. Moreover, reducing the time of phase change of the PCM will allow the R-STEG to produce more energy. In addition, improvement of thermoelectric material properties can increase the energy conversion efficiency. This work introduced a new route for solar energy generation in which the optical properties of the PCM are utilized in addition to energy storage.

## Methods

### Fabrication of refraction-changed STEG

The refraction-assisted STEG can be divided into three components: the dome-shaped PDMS lens, the PDMS container of the PCM, and the TEG module. The radius of the dome-shaped PDMS lens was 25 mm. The overall size of the PDMS container was 80 mm × 80 mm × 20 mm, with the PDMS container having an internal radius of 25 mm. Supplementary Fig. S3 summarizes the fabrication steps for a refraction-assisted STEG. The fabrication begins with production of the PDMS dome-shaped lens. The 10:1 PDMS/curing agent mixture (Sylgard 184, Dow Corning) was poured into the bowl-shaped PMMA mold. After curing for at least 3 h at 65 °C, the PDMS was peeled from the mold, as shown in Fig. S3(a). The PDMS container was fabricated in the same manner as described above. Then, 10 g of the liquid PCM (n-octadecane, Sigma-Aldrich) was poured into the fabricated PDMS container, as shown in Fig. S3(b). After hardening the PCM at room temperature, a wave-selective solar absorber (mirotherm, Alanod-solar) with dimensions 12.5 mm × 12.5 mm was attached under the PDMS container, as shown in Fig. S3(c). The TEG module (TEC1-12715, Thermonamic electronics) had 127 thermocouples with dimensions of 50 mm × 50 mm. Finally, the fabricated elements were combined in order, as shown in Fig. S3(d).

### Experimental setup

The experimental setup comprised a solar simulator based on tungsten halogen lamps, a digital oscilloscope (U1640B, Agilent), a solar power meter (TES-132, Lutron), and a thermometer (TM-947SD, Lutron Electronics). The refraction-assisted STEG and typical STEG were illuminated under controlled radiation using the solar simulator and cooled by natural convection. To measure the temperature change of the hot and bottom sides, one thermocouple was connected to the wave-selective solar absorber and another connected to the cold side. The power output was measured at a load resistance of 1.5 Ω.

## Additional Information

**How to cite this article**: Kim, M.-S. *et al*. Refraction-Assisted Solar Thermoelectric Generator based on Phase-Change Lens. *Sci. Rep*. **6**, 27913; doi: 10.1038/srep27913 (2016).

## Supplementary Material

Supplementary Information

Supplementary Video S1
