# Supplementary material for: Refraction-Assisted Solar Thermoelectric Generator based on Phase-Change Lens
Source: Sci Rep. 2016 Jun 10;6:27913. doi: 10.1038/srep27913 (PMC4901302; doi:10.1038/srep27913)
Supplement: Supplementary Information [file srep27913-s1.pdf]

Supplementary information

Refraction-Assisted Solar Thermoelectric Generator based on Phase-Change lens

Myoung-Soo Kim, Min-Ki Kim, Sung-Eun Jo, Chulmin Joo, and Yong-Jun Kim*

Department of Mechanical Engineering, Yonsei University, 50 Yonsei-ro, Seodaemun-Gu, Seoul, Republic of Korea.

Corresponding author: Yong-Jun Kim

E-mail: yjk@yonsei.ac.kr

Temperature of the Hot Side of Refraction-Assisted STEG

In the refraction-assisted STEG shown in Fig. 3, we assumed that the heat flux is constant. The heat flows $\dot{Q}_{sun}$ and $\dot{Q}_{PCM}$ are currents that depend on an energy source, and the temperatures of each point T_{sun}, $T_{sensible}$, T_{latent}, and T_h are voltages. The thermal resistances R_{PCM}, $R_{absorber}$, R_{TEG}, $R_{cold\ side}$, and $R_{air\ convection}$ are resistances that connects in series. Voltage gains of the amplifier are denoted with G_1 and G_2. The heat flow and hot side temperature of the TEG vary according to the operating mechanism described in the manuscript.

i) Solar energy generation with energy storage based on the phase change of the PCM in daytime.

The heat transfer rate is given by

$$\dot{Q}_{sun} = \frac{G_1 T_{sun} - T_h}{R_{PCM} + R_{absorber}} \tag{S1}$$

Following Equation (S1), the hot side temperature of the TEG is

$$T_h = G_1 T_{sun} - \dot{Q}_{sun}(R_{PCM} + R_{absorber}) \tag{S2}$$

ii) Assistance of the phase-changing lens to refocus the solar energy in the daytime.

The heat transfer rate is given by

$$\dot{Q}_{sun} = \frac{G_1 G_2 T_{sun} - T_h}{R_{PCM} + R_{absorber}} \tag{S3}$$

Following Equation (S3), the hot side temperature of the TEG is

$$T_h = G_1 G_2 T_{sun} - \dot{Q}_{sun}(R_{PCM} + R_{absorber}) \tag{S4}$$

iii) Energy generation via release of sensible and latent heat stored in the liquid PCM.

The heat transfer rate is given by

$$\dot{Q}_{PCM} = \frac{T_{sensible\ or\ latent} - T_h}{R_{PCM} + R_{absorber}} \tag{S5}$$

Following Equation (S5), the hot side temperature of the TEG is

$$T_h = T_{sensible\ or\ latent} - \dot{Q}_{sun}(R_{PCM} + R_{absorber}) \tag{S6}$$

The equivalent thermal resistance of the PCM is

$$R_{PCM} = \frac{h_{PCM}}{\pi (r_{PCM})^2 k_{PCM}} \tag{S7}$$

where k_{PCM} is the thermal conductivity of the PCM and h_{PCM} and r_{PCM} are the height and radius of the PCM, respectively.

Heat Capacity of the PCM

The equivalent thermal capacitance of the PCM is calculated as

$$C_{PCM} = \pi (r_{PCM})^2 h_{PCM} C_{app} \rho_{PCM} \tag{S8}$$

where ρ_{PCM} is the density of the PCM and C_{app} is the apparent heat capacity of the PCM. The C_{app} is defined as

$$C_{app} = \begin{cases} C_p & (T_{PCM} \leq T_{melt}) \\ C_p + \dfrac{\Delta H}{\mathrm{d}T} & (T_{melt} \leq T_{PCM} \leq T_{melt} + \mathrm{d}T) \\ C_p & (T_{PCM} \geq T_{melt} + \mathrm{d}T) \end{cases} \tag{S9}$$

where C_p is the specific heat capacity of the PCM, T_{PCM} is the temperature of the PCM, ΔH is the latent heat of melting, dT is the temperature range over which the PCM melts, and T_{melt} is the melting point of the PCM.[S1]

Efficiency of the Refraction-Assisted STEG in the Daytime

Solar radiation is absorbed by the wave-selective absorber through the optical lens. The absorbed energy collected in the absorber through the optical lens (the dome-shaped PDMS lens and the liquid PCM lens) is the sum of the thermal radiation loss from the absorber, the heat transferred to the TEG, and the conduction loss to the ambient environment. We make the following assumptions: (i) the absorber temperature is equivalent to the hot side of the TEG (T_h); (ii) the absorber is at a uniform temperature T_h; (iii) the thermal contact resistances in the R-STEG are negligible; (iv) the conduction losses to the ambient environment are negligible; (v) the ambient temperature (T_{amb}) is equivalent to the cold side of the TEG (T_c).

A steady-state energy balance on the absorber yields,

$$\tau_{pp}\alpha C_{opt}q_i A = Q_{te} + A\varepsilon\sigma(T_h^4 - T_{amb}^4) \tag{S10}$$

where Q_{te} is the heat transferred to the TEG, τ_{pp} is the total transmittance of the PDMS and the liquid PCM, α is the absorptance, ε is the effective emittance of the wavelength-selective solar absorber, σ_{sb} is the Stefan-Boltzmann constant, C_{opt} is the optical concentration of the dome-shaped PDMS lens and the liquid PCM lens, T_h is the temperature of the hot side, T_{amb} is the ambient temperature, and q_i is the incident solar flux.[S2]

Based on the energy balance analysis, the optical lens thermal absorber efficiency (η_{opth}) can be written as

$$\eta_{opth} = \frac{Q_{te}}{Q_{in}} = \tau\alpha - \frac{\varepsilon\sigma_{sb}(T_h^4 - T_{amb}^4)}{C_{opt}q_i} \tag{S11}$$

where $Q_{in} = q_i A$ is the sum of the incident sunlight energy on the dome-shaped PDMS lens; here the solar irradiance is q_i and the area of the lens is A.

The daytime efficiency of the refraction-assisted STEG with the liquid PCM lens ($\eta_{\mathrm{R\text{-}STEG}}$) can be approximately expressed as the product of the optical lens thermal absorber efficiency (η_{opth}) and the thermoelectric device's efficiency (η_{TEGs}):

$$\eta_{R-STEG} = \eta_{opth}\eta_{TEGs} \approx \left[\tau\alpha - \frac{\varepsilon\sigma_{sb}(T_h^4 - T_{amb}^4)}{C_{opt}q_i}\right]\eta_{TEGs} \tag{S12}$$

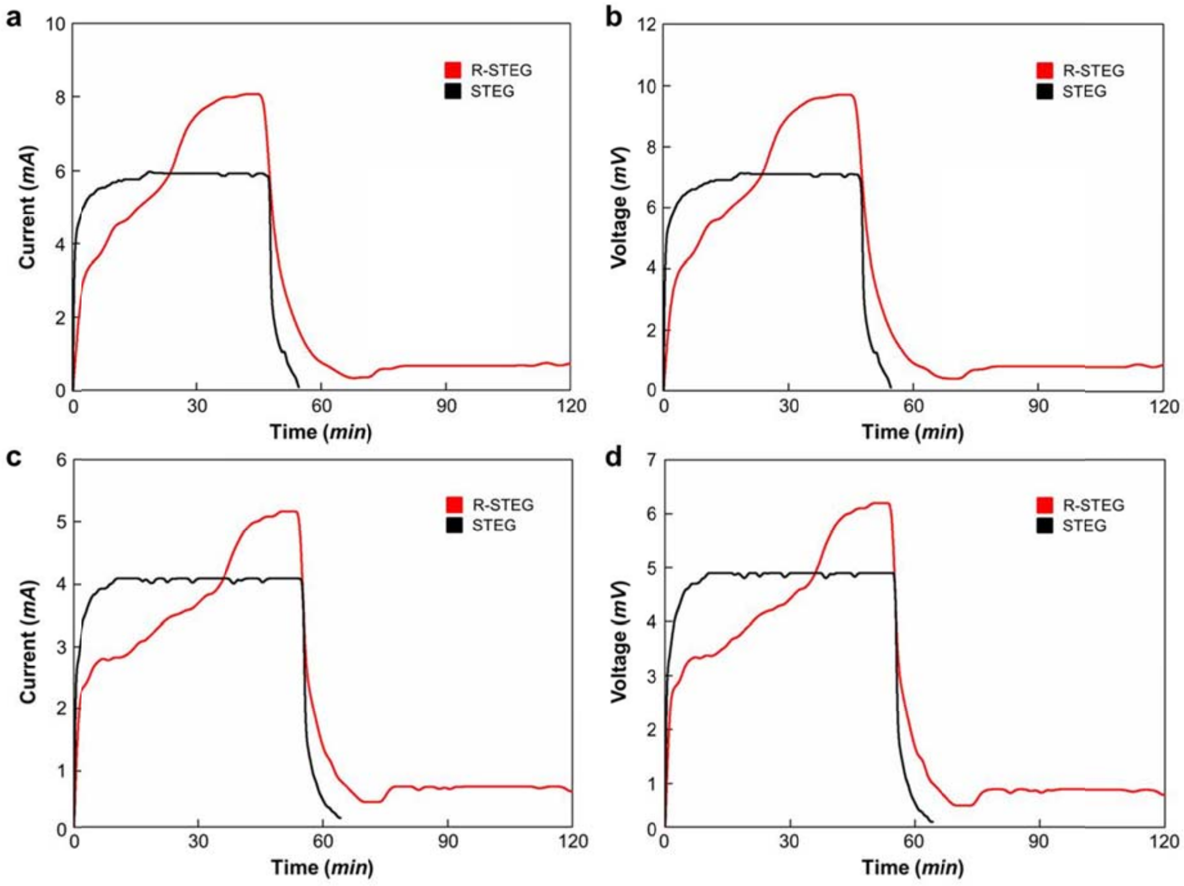

Figure S1. Comparisons of the electrical measurements of the refraction-assisted STEG (R-STEG) and typical STEG (STEG). (a) Currents at incident solar radiation flux of 1.5 kW m^{-2}. (b) closed-circuit voltages at incident solar radiation flux of 1.5 kW m^{-2}. (c) Currents at incident solar radiation flux of 1 kW m^{-2}. (d) Closed-circuit voltages at incident solar radiation flux of 1 kW m^{-2}.

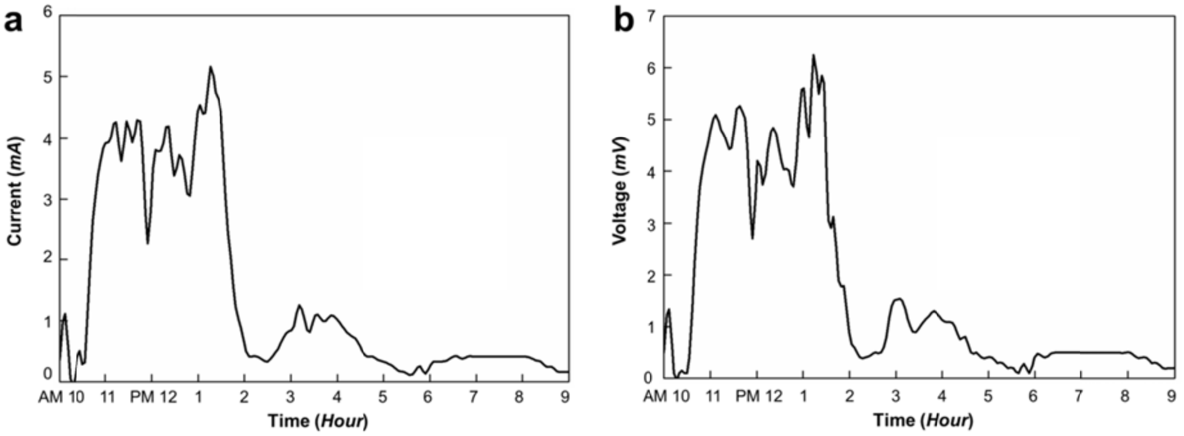

Figure S2. Demonstration of the refraction-assisted STEG (R-STEG) in an open environment. (a) Closed-circuit current, (b) voltage along the experimental timeline.

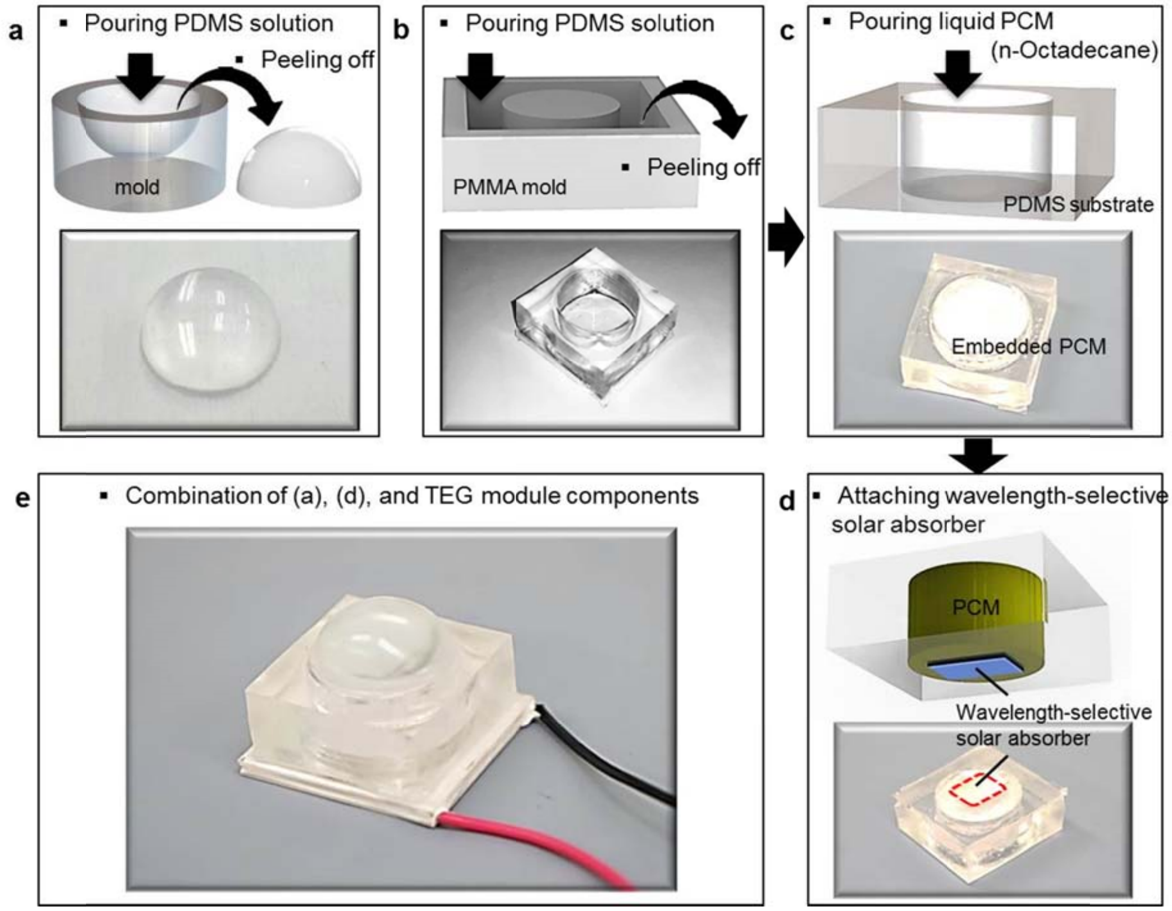

Figure S3. Schematic illustration of the refraction-assisted STEG fabrication process.

(a, b) Pouring the PDMS/curing agent mixture into the PMMA mold; curing and removing the PDMS from the mold. (c) Pouring 5 g of liquid PCM into fabricated PDMS container. (d) Attaching a wave-selective solar absorber under the PDMS container after the PCM hardens at room temperature. (e) Final combination of PDMS lens, PCM cylinder, PDMS container, wavelenth-selective solar absorber, and TEG module components.

Supplementary movie S1

Crystalline structure changes of the phase change material (n-octadecane) according to phase-change. The refractive index and transmittance of the phase change material vary with crystalline structure change.

References:

S1 Jo, S.-E., Kim, M.-S., Kim, M.-K. & Kim, Y.-J. Power generation of a thermoelectric generator with phase change materials. Smart Materials and Structures **22**, 115008 (2013).

S2 Kraemer, D. et al. High-performance flat-panel solar thermoelectric generators with high thermal concentration. Nature materials **10**, 532-538 (2011)
